# Perineal rectosigmoidectomy for rectal prolapse—the preferred procedure for the unfit elderly patient? 10 years experience from a UK tertiary centre

**DOI:** 10.1007/s10151-019-02100-z

**Published:** 2019-11-13

**Authors:** M. Alwahid, S. R. Knight, H. Wadhawan, K. L. Campbell, D. Ziyaie, S. M. P. Koch

**Affiliations:** Ninewells Hospital and Medical School, Dundee, DD1 9SY UK

**Keywords:** Rectal prolapse, Perineal rectosigmoidectomy, Altemeier, Age

## Abstract

**Background:**

Rectal prolapse is a disease presentation with a prevalence of about 1%, mainly affecting older women. It usually presents with symptoms of rectal mass, rectal bleeding, fecal incontinence or constipation, with patients frequently feeling socially isolated as a result. Perineal rectosigmoidectomy is associated with lesser morbidity and mortality than the abdominal procedure, but with a much higher recurrence rate. Therefore, this technique is mainly suitable for the frail elderly patient. Specific outcomes in an elderly population have been described in only a few studies. We evaluated the morbidity, mortality, recurrence rate and functional results after this procedure related to age.

**Methods:**

All patients who underwent a perineal rectosigmoidectomy over a 10-year period in two tertiary referral centers were included in the study. American Society of Anesthesiology (ASA) grade, pre- and postoperative symptoms, pathology-reported post-fixation specimen length, length of in-patient stay, 30-day morbidity/mortality, and recurrence were measured.

**Results:**

A total of 45 patients underwent a perineal rectosigmoidectomy. Forty-three (95%) were female, with a median age of 82.0 years (IQR 70.5–86.5), ASA grade III and median follow-up of 20 months (range 8.5–45.5 months). Half of the cohort was over 80 years old. Significant symptomatic relief was achieved, predominantly the resolution of rectal mass (8.9% vs. 60.0% preoperatively), fecal incontinence (15.6% vs. 46.7%) and constipation (4.4% vs. 26.7%). The median length of stay was 6 days, while morbidity occurred in 14 patients (31.1%) and recurrence occurred in 6 patients (13%). There were no deaths within 30 days of the procedure and outcomes were comparable in the < 80 and ≥ 80 age group.

**Conclusions:**

Perineal rectosigmoidectomy is safe for older patients with greater comorbidities resulting in good functional results and is associated with low morbidity and mortality.

## Introduction

Rectal prolapse is a disease presentation mainly affecting older women, with a prevalence of up to 1% in adults over 65 years old [1]. The main reported symptoms are a feeling of a rectal mass, and fecal incontinence in 35–100% of cases [2].

It can be associated with significant morbidity including rectal bleeding, obstructive defecation, pain and possible strangulation [3]. Surgical intervention is usually required to correct the prolapse, preferably with an abdominal procedure, that is associated with a lower recurrence rate and better long-term functional outcome [2, 4, 5]. The laparoscopic technique is favorable compared to an open technique. It is associated with lower costs due to a shorter hospital stay and faster patient recovery [6]. However, this can be a major operation in elderly patients with multiple comorbidities and frailty, as well as in infirm younger patients. In these patients with significant comorbidities that pose an anesthetic risk, precluding a general anesthesia, perineal rectosigmoidectomy under spinal anesthesia is favored due to a lower operative morbidity rate and quicker recovery compared to the intra-abdominal approach [7]. Unfortunately, the perineal approach is associated with an increased rate of recurrence [8, 9], and a poorer functional outcome [2, 10]. Evidence of its efficacy and safety specifically in the 80 and above age group is currently limited [11–13].

There are two main perineal-based approaches for rectal prolapse, the Delorme and Altemeier procedures [14]. Both are associated with a low mortality rate, but a high recurrence rate [15, 16]. The Altemeier procedure is a rectosigmoidectomy, which involves a full-thickness resection of the rectum starting around 1 cm proximal to the dentate line and often extends to the sigmoid colon, together with the redundant anterior peritoneum. A coloanal anastomosis is then performed with either absorbable interrupted sutures or a stapler device [17]. The original Altemeier procedure also entails restoration of the pelvic floor, by means of an additional levatorplasty [18]. Delorme’s procedure involves a circular excision of the mucosa and plication of rectal wall muscle [15, 16].

The aim of this retrospective study was to determine the morbidity, mortality and recurrence rates in patients undergoing perineal rectosigmoidectomy for rectal prolapse in two tertiary referral centers, comparing results in patients < 80 and ≥ 80 years old.

## Materials and methods

All patients who had a perineal-based rectosigmoidectomy between 2004 and 2014 in two institutions within National Health Service (NHS) Tayside, Scotland were identified retrospectively using electronic operative records.

The technique has been described before [18]. Since it was not routinely reported in the operative record whether a levatorplasty was performed and in some cases, operative notes were missing, we could not include this in the data analysis.

In all patients, the peritoneal cavity was breached to free the pouch of Douglas from any attendant enterocele so as to ensure small bowel reduction. Procedures were performed by Consultant Colorectal Surgeons at either Ninewells Hospital, a university teaching hospital in Dundee, or Perth Royal Infirmary, a district general teaching hospital in Perth. All surgeons were experienced colorectal surgeons with expertise in the procedure, three of four with experience of over 10 years as a consultant surgeon. The study was performed between 2004 and 2014 by four colorectal surgeons, with most procedures performed by two colorectal surgeons further sub-specialized in pelvic floor surgery. The study was registered locally with the Research and Development department by means of Caldicott approval.

Case notes were retrospectively reviewed, with baseline patient characteristics, symptoms at presentation, operative time, length of specimen post-fixation in formalin, length of in-patient stay, 30-day morbidity and mortality, the presence of postoperative symptoms and evidence of recurrence recorded at clinic follow-up. No patients were excluded from the analysis. Outcomes for the whole cohort, together with subgroup analysis for age groups < 80 and ≥ 80 years old were also performed.

### Statistical analysis

Data analysis was performed using the statistical package R v3.3.1 (http://www.r-project.org/) [19]. Differences between categorical data were analyzed using Fisher’s exact test, while continuous data were analyzed using the Mann–Whitney *U* test. For all data, the median value is provided together with interquartile range (IQR) except where otherwise stated. Mean values are provided with standard deviation (SD). *p* ≤ 0.05 was considered statistically significant.

## Results

Over a 10-year period, 45 patients underwent a rectosigmoidectomy procedure for their rectal prolapse. The majority were female (male:female ratio 2:43) and median patient age was 82.0 years (IQR 70.5–86.5) with a mean age of 78 years (SD 13) and with half of the cohort (*n* = 23) aged 80 or over. The cohort contained a median American Society of Anesthesiologists (ASA) grade of III, consisting of 18 patients with an ASA grade III or IV. The median body mass index (BMI) was 23.0 kg/m^2^ (IQR 20.1–25.0 kg/m^2^). The patient characteristics are detailed in Table 1. Characteristics of patients aged < 80 and ≥ 80 years old were similar.

**Table 1:** Patient characteristics

	Whole cohort	Age < 80 years	Age ≥ 80 years	*p* value
Number of patients	45	22	23	na
Sex (M:F)	2:43	1:21	1:22	1.000
Age (years)	82.0 (70.5–86.5)	71.0 (63.0–75.0)	86.0 (85.0–91.0)	na
ASA class	3.0	3.0	3.0	0.802
BMI kg/m^2^	23.0 (20.5–25.0)	23.5 (20.5–24.5)	23.0 (21.5–25.0)	**0.042**
Operation time (mins)	105.0 (75.0–120.5)	110.0 (75.0–130.0)	97.0 (71.3–120.3)	0.753
Length of specimen (cm)	8.9 (6.9–13.6)	9.5 (7.0–14.3)	8.0 (6.5–13.8)	0.773
Length of stay (days)	6.0 (4.5–11.5)	5 (5.0–13.5)	6.5 (3.5–11.0)	0.775
Follow-up (months)	20.0 (8.5–45.5)	25 (8.5–46.5)	17.5 (8.5–31.0)	0.391

Values stated are median and IQR in parentheses

*p* value in bold means a significant difference

*na* not applicable, *ASA* American Society of Anesthesiologists, *BMI* body mass index

At presentation, the most frequent patient-reported symptom was the sensation of a rectal mass (*n* = 27, 60.0%), with additional symptoms of fecal incontinence (*n* = 21, 46.7%), constipation (*n* = 12, 26.7%), rectal bleeding (*n* = 9, 20.0%) and painful defecation (*n* = 8, 17.8%; Table 2).

**Table 2:** Symptoms at presentation

(a) Whole cohort			
	Preoperative symptoms Number of patients (%)	Postoperative symptoms Number of patients (%)	*p* value
Rectal mass	27 (60.0)	4 (8.9)	**< 0.001**
Painful defecation	8 (17.8)	2 (4.4)	0.080
Fecal incontinence	21 (46.7)	7 (15.6)	**0.002**
Rectal bleeding	9 (20.0)	3 (6.7)	0.118
Constipation	12 (26.7)	2 (4.4)	**0.007**

(b) Cohort separated by age						
	Preoperative symptoms Number of patients (%)		*p* value	Postoperative symptoms Number of patients (%)		*p* value
	Age < 80 years	Age ≥ 80 years		Age < 80 years	Age ≥ 80 years	
Rectal mass	14 (63.6)	13 (56.5)	0.763	3 (13.6)	1 (4.3)	0.346
Painful defecation	5 (22.7)	3 (13.0)	0.459	2 (9.1)	0	0.233
Fecal incontinence	7 (31.8)	14 (60.9)	0.074	2 (9.1)	5 (21.7)	0.414
Rectal bleeding	5 (22.7)	4 (17.4)	0.722	2 (9.1)	1 (4.3)	0.607
Constipation	7 (31.8)	5 (21.7)	0.513	0	2 (8.7)	0.488

*p* values in bold mean a significant difference

The median operation time was 105.0 min (IQR 75.0–120.5 min), while specimen length measured 8.9 cm (IQR 6.9–13.6 cm).

Median length of stay was 6.0 days (IQR 4.5–11.5 days) with three patients staying longer than 30 days (32, 39 and 61 days, respectively). In patients aged ≥ 80, operation time was 97.0 min (IQR 71.3–120.3 min) and median length of stay was 6.5 days (IQR 3.5–11.0 days), comparable to the patients < 80 years old. Significant reductions in patient-reported symptoms were observed for the majority of categories (Table 2a), feeling of rectal mass 8.9% vs. 60% pre-operatively (*p* = < 0.001), fecal incontinence 15.6% vs. 46.7% (*p* = 0.002)  and constipation 4.4% vs. 26.7% (*p* = 0.007), with reductions comparable between the two age groups (Table 2b). Postoperative complications were observed in 14 patients (31.1%), two-thirds of which were local complications (Table 3). The overall complication rate was lower in the ≥ 80 group (*n* = 5; 22.7%), however, not significantly different from that in the < 80 group (*n* = 9; 42.9%, *p* = 0.208).

**Table 3:** Postoperative complications following perineal rectosigmoidectomy

	Number of patients (%)		
	Whole cohort	Age < 80 years	Age ≥ 80 years
General complications			
Pneumonia	2 (4.4)	0	2 (9.0)
Transient ischemic attack	1 (2.2)	1 (4.5)	0
Incarcerated femoral hernia	1 (2.2)	0	1 (4.5)
Local complications			
Anastomotic leak	3 (6.7)	2 (9.1)	1 (4.5)
Pararectal abscess	1 (2.2)	0	1 (4.5)
Hemorrhage	2 (4.4)	2 (9.1)	0
Postoperative diarrhea	2 (4.4)	2 (9.1)	0
Rectal ulcer	2 (4.4)	2 (9.1)	0
Total complications	14 (31.1)	9 (42.9)	5 (22.7)
Patients requiring reoperation	3 (6.7)	2 (9.1)	1 (4.5)
Death	0	0	0

Three patients with an anastomotic leak required a return to theater, with one having a laparotomy and washout without takedown of the coloanal anastomosis, while the other two had an end colostomy formed as a definitive procedure. Only one of these patients was over 80 years old. There was no relationship between the length of the resected specimen or ASA grade and the anastomotic leak rate. At the end of the study period (10 years), there were no deaths related to the procedure. However, 13 patients in total, 7 in the ≥ 80 age group, had died of unrelated causes. The median follow-up period was 20 months (range 8.5–45.5 months) and similar in patients < 80 and ≥ 80 years old (Table 1).

Recurrence occurred in six patients (13%) with a mean time until recurrence of 1.5 years (SD 0.5). Four patients underwent a further procedure: two had a further perineal rectosigmoidectomy, one a laparoscopic suture rectopexy without mesh and one a perineal stapled prolapse resection; (Table 4). The resected specimen length was not associated with a risk of recurrence (*p* = 0.920). The recurrences were divided amongst three of the four surgeons, none of them exceeding a recurrence rate of 20%.

**Table 4:** Outcomes after perineal rectosigmoidectomy

	Number of patients (%)		
	Whole cohort	Age < 80 years	Age ≥ 80 years
30-day mortality	0	0	0
Reoperation	3 (6.7)	2 (9.1)	1 (4.5)
Recurrence	6 (13.3)	4 (18.2)	2 (9.0)
Second procedure	4 (8.9)	3 (13.6)	1 (4.5)

The recurrence rate in patients ≥ 80 years was 8.7% (*n* = 2), compared to 18.2% (*n* = 4) in the < 80 group.

## Discussion

Our study evaluated the safety and effectiveness of the perineal rectosigmoidectomy procedure for rectal prolapse in an elderly population. The mean age of 78 years in our study population was higher than any previously published series for the descried technique (Table 5), except for the small study of Johansen [12], demonstrating that the procedure can be performed successfully in patients aged above 80 years old with a relatively low complication and recurrence rate comparable to patients under 80 years of age. Cirocco et al. reported 39 patients over 80 years old, but did not specifically compare the > 80 group with the < 80 group [11]. Ram reported 14 patients with a mean age of 80 years, but they all had perineal stapled prolapse resection, so the results were slightly different [20].

**Table 5:** Literature overview for perineal rectosigmoidectomy

Author	*N*	Study design	Age (years)	Mortality (%)	Morbidity (%)	Leak rate (%)	Incontinence		Recurrence (%)	Levatorplasty	Follow-up (months)
							Preop	Postop			
Altemeier et al. [18]	106	Retrospective	62.2^b^	0	24.5	2.8	NR	NR	2.8	+	228^b^
Watts et al. [28]	33	Retrospective	NR	0	NR	NR	NR	NR	0	NR	23^b^
Williams et al. [13]	114	Retrospective	78^a^	0	12	0.9	59	32	10	+-	12^a^
Johansen et al. [12]	20	Prospective	82^b^	5	NR	NR	NR	NR	0	+	26^b^
Deen et al. [29]	10	Retrospective	NR	0	NR	NR	NR	20	10	+	18^b^
Ramanujam, et al. [10]	72	NR	NR	0	NR	NR	NR	66.7i	6	+	120^b^
Agachan et al. [8]	32	Retrospective	75^b^	NR	1	3.3	NR	NR	13	NR	30^b^
Kim et al. [36]	183	Retrospective	64^a^	NR	14	NR	NR	NR	16	NR	47^b^
Zbar et al. [26]	80	Retrospective	69^b^	0	2	2	5	0	4	some	22^a^
Habr-Gama et al. [24]	44	Retrospective	76^b^	0	9	NR	NR	14	7.1	+	49^b^
Hammond et al. [9]	48	Retrospective	60.8	0	NR	NR	NR	NR	17	–	39^b^
Glasgow et al. [27]	103	Retrospective	75^a^	0.9	8.5	4	75.5	41.5	8.5	68-86%	21^a^ 36^b^
Kim et al. [25]	38	Prospective	75^a^	2.6	18.4	0	72	72	2.6	12/38	24^a^
Cirocco [11]	103	Retrospective	69^b^	0	14	0	47	7	0	+	43^b^
Ris et al. [31]	60	Prospective	77	1.6	11.6	1.7	NR	62i	14	21/60	48^b^
Mik et al. [35]	45	Prospective	67	0	19.1	8.9	NR	NR	14.8	18/45	32^b^
Current study	45	Retrospective	82^a^78^b^	0	31.1	6.7	46.7	15.6	13	NR	20^b^

*NR* not recorded, *i* improvement

^a^Median

^b^Mean

The most significant postoperative complication was anastomotic leak. The rate of anastomotic leak demonstrated in our study (6.7%) was similar to those previously published (Table 5). Three patients with an anastomotic leak required a return to theater, with one having a laparotomy and washout without takedown of the coloanal anastomosis, while the other two had an end colostomy formed as a definitive procedure. The advantage of a rectosigmoidectomy is the presence of a coloanal anastomosis. When there is a leak in the anastomosis, naturally it drains through the anus. The reasoning for a specific technique chosen in case of reoperation for an anastomotic leak was not stated in the notes.

There were no deaths within 30 days of the procedure. Furthermore, only one patient over 80 years developed an anastomotic leak, so that the leak rate was 4.3% in this subgroup.

Recurrence rate in our cohort was 13.3%, occurring after a mean time of 1.5 years (SD 0.5) after the procedure, with only four patients requiring a second operation. Historically recurrence rates as high as 50–60% have been reported after perineal rectosigmoidectomy [14, 16, 21], but the actual rate is between 0 and 20% [11, 22–29], and our findings support current estimates of recurrence (Table 5). In addition, in patients aged 80 or over the recurrence rate was slightly lower (8.7%), with both occurring within the first year post-procedure.

Risk factors for recurrence are the absence of levatorplasty [23], short specimen length and perineal stapled prolapse resection [30]. Our study demonstrated that specimen length (median length 8.9 cm) did not impact upon recurrence rates. A multivariate analysis has shown a fourfold increase in recurrence rate with specimens shorter than 7 cm [30]. However, other studies have failed to demonstrate such an association [31, 32], and high variance within the study by Kim et al. limits conclusions. Furthermore, a smaller resection may provide greater symptomatic relief, with postoperative fecal incontinence improved with shorter specimen lengths [31], and may also be related to a less severe prolapse in these patients.

In all patients, the peritoneal cavity was breached to free the pouch of Douglas from any attendant enterocele so as to ensure small bowel reduction. It is argued that the incomplete mobilization of the rectum from the peritoneal pouch of the Douglas may explain the high rate of relapse in some reports [33].

There are institutions that advocate levatorplasty in addition to the perineal rectosigmoidectomy [11, 18, 24, 29]. This was first described by Cohn in 1942. The levatorplasty was routinely performed by Altemeier beginning with his first published series in 1952 [34]. Authors note that it is easily performed and results in a beneficial effect on postoperative continence [13, 23]. Mik et al. state that an anterior levatoroplasty during the Altemeier procedure should be performed in the group of female patients with co-existing fecal incontinence to improve anal sphincter function [35].

Williams et al., showed that in 26/56 patients with rectosigmoidectomy alone fecal incontinence improved, compared to 10/11 patients with additional levatorplasty [13].

Some studies, however, do not show a benefit of the levatorplasty. In a study by Kim et al., a levatorplasty was performed in 12/38 patients. There was no significant difference between patients with and without levatorplasty in terms of continence, constipation and quality of life [25]. Due to the different study designs (Table 5), no conclusions can be drawn as to whether a levatorplasty results in better continence (Table 5).

Some authors state that an additional levatorplasty is the main reason for the improvement in recurrence rates and the subsequent resurgence of the operation [11]. Abdominal operations do not provide access for levatorplasty, but still the recurrence rates are much lower. Cirocco suggests that it is time to reconsider a combined abdominal and perineal approach [7].

The possibility of postoperative evacuatory disorder after additional levatorplasty should be kept in mind. Zbar et al. reported that an anterior levatorplasty can be performed. In that series, 13% of patients complained of postoperative difficulty evacuating, which is not normally reported after perineal rectosigmoidectomy and is perhaps consequent to an overly tight levatorplasty [26].

In the current study, levatorplasty was not routinely performed. The exact number of levatorplasty procedures was not known; therefore, this was not been used in data analysis.

A prospective study randomizing between patients with or without levatorplasty with quality of life assessment would give a clearer answer.

The effect of perineal rectosigmoidectomy on constipation has been explored previously [14, 25, 27, 36]. We found an 83% improvement in 26.7% of patients who reported constipation preoperatively. In another prospective study, by Kim et al., there was patient-reported constipation preoperatively in 70% of 29 patients which persisted postoperatively in 62%. However, in the same study, the mean (SD) Cleveland Clinic Constipation Score improved from 10.21 (6.76) before surgery to 3.58 (3.26) after Altemeier’s procedure *(p* < 0.001) [25].

The current study also demonstrated patient-reported fecal incontinence improved in two-thirds of the patients, from 46.7% preoperatively to 15.6% postoperatively, which is comparable to other series after rectosigmoidectomy [14, 22, 31, 32].

In addition, as the present study is the only study that specifically reports outcomes following perineal rectosigmoidectomy in patients ≥ 80 years old, further studies within this particular age group are required to validate our results.

As yet no study has formally investigated the relationship between the method of rectal prolapse repair; abdominal rectopexy, perineal rectosigmoidectomy or Delorme’s procedure; and changes in quality of life. Increasingly, quality of life is being considered as an important marker of surgical outcome [37–39], and in a potentially frail population, it would be of interest to explore the effect of both perineal and abdominal procedures on patients’ quality of life as well as physical symptoms. Kim et al. in a prospective case series found perineal rectosigmoidectomy significantly improved patient-reported quality of life in nearly all domains. Only self-care was not significantly better, indicating that inability to wash or dress is not improved by prolapse correction; however, only 37 patients completed pre- and postoperative questionnaires [39].

Over 100 operations to repair rectal prolapse have been described [22], with the 2 main perineal approaches being perineal rectosigmoidectomy and Delorme’s procedure. Studies on Delorme’s procedure report high recurrence rates [16, 40–42]; however, symptomatic improvement appears to be similar between the two techniques [16, 22, 31, 40]. Lower recurrence rates with perineal rectosigmoidectomy, as demonstrated within our cohort, suggest possible advantages for an elderly age group compared to the Delorme’s procedure for rectal prolapse. Furthermore, our results show that a perineal rectosigmoidectomy confers a mortality advantage compared to abdominal approaches for rectal prolapse, with recurrence rates and symptomatic improvement, that may be equivalent [2, 8–10]. However, in very frail patients in whom the risk of resection is considered to be too high, a Delorme’s procedure may still be the procedure of choice [7, 41, 42].

Laparoscopic ventral mesh rectopexy is currently the preferred procedure in external rectal prolapse in Europe. The American guidelines are less convinced about the benefits of ventral mesh rectopexy, because there are not many studies with long-term results concerning recurrence and mesh-related problems [43]. This procedure can be safely performed in fit patients, even if they are elderly. Wijffels et al. showed in 2011 that even in patients with a high ASA grade, the laparoscopic procedure could be performed [44]. In elderly patients with significant co-morbidities that pose an anesthetic risk, precluding a general anesthesia, perineal rectosigmoidectomy under spinal anesthesia is favored. In addition, staying outside of the abdominal cavity with a transanal approach such as the Altemeier or Delorme procedure in ‘infirm’ non-elderly, perhaps institutionalized, patients with prolapse, with significant co-morbidities who are not good candidates for a major abdominal operation may be considered regardless of age. This seems preferable to the patient being turned away by the surgeon.

Perineal rectosigmoidectomy is also a treatment option for patients, who have no abdominal access due to previous extensive abdominal surgery.

This is in accordance with the Dutch and Italian guidelines for external rectal prolapse [33, 45].

Limitations of the present study include the absence of a comparison to another procedure, such as the Delorme’s procedure or abdominal repair, which can help to assess the relative benefits and risks of each procedure in a particular population.

Furthermore, perioperative functional measurement of anal sphincter muscles or resting pressures, used in other series, was not available [31], and due to the study’s retrospective nature, the measurement of quality of life was not possible.

## Conclusions

Perineal rectosigmoidectomy is safe and effective in older patients with co-morbidities. The procedure has good functional results and is associated with low morbidity and mortality.

A randomized prospective study comparing perineal rectosigmoidectomy to laparoscopic rectopexy in the elderly frail patients over 80 years of age would be of great value.
